# Protein palmitoylation is involved in regulating mouse sperm motility via the signals of calcium, protein tyrosine phosphorylation and reactive oxygen species

**DOI:** 10.1186/s40659-024-00580-4

**Published:** 2025-01-15

**Authors:** Yuping Xiong, Chenchen Yi, Haixia Zheng, Ya Ni, Yamei Xue, Kun Li

**Affiliations:** 1 School of Pharmacy, Hangzhou Medical College, Hangzhou, Zhejiang China; 2 School of Basic Medical Sciences and Forensic Medicine, Hangzhou Medical College, Hangzhou, Zhejiang China; 3 Department of Obstetrics and Gynecology, Assisted Reproduction Unit, Sir Run Run Shaw Hospital, School of Medicine, Zhejiang University, Hangzhou, Zhejiang China

**Keywords:** Protein palmitoylation, Sperm motility, Protein tyrosine phosphorylation, Calcium, Reactive oxygen species, Asthenozoospermia

## Abstract

**Background:**

Protein palmitoylation, a critical posttranslational modification, plays an indispensable role in various cellular processes, including the regulation of protein stability, mediation of membrane fusion, facilitation of intracellular protein trafficking, and participation in cellular signaling pathways. It is also implicated in the pathogenesis of diseases, such as cancer, neurological disorders, inflammation, metabolic disorders, infections, and neurodegenerative diseases. However, its regulatory effects on sperm physiology, particularly motility, remain unclear. This study aimed to elucidate the mechanism by which protein palmitoylation governs sperm motility.

**Methods:**

Protein palmitoylation in situ in mouse sperm was observed using innovative click chemistry. Sperm motility and motion parameters were evaluated using a computer-assisted sperm analyzer (CASA) after treatment with 2-bromopalmitic acid (2BP), a specific inhibitor of protein palmitoylation. Protein palmitoylation levels were confirmed by the acyl-biotin exchange (ABE) method. The interplay between protein palmitoylation, protein tyrosine phosphorylation, and intracellular calcium was investigated using Western blotting, ABE method, and fluorescent probes. The regulation of reactive oxygen species was also examined using fluorescent probes.

**Results:**

Localized patterns and dynamics of protein palmitoylation in distinct sperm regions were revealed, including the midpiece, post-acrosomal region, acrosome, and head. Alterations in protein palmitoylation in sperm were observed under in vitro physiological conditions. Treatment with 2BP significantly affected sperm motility and motion parameters. The study revealed interactions between protein palmitoylation, including heat shock protein 90, and protein kinase A/protein kinase C-associated protein tyrosine phosphorylation and intracellular calcium. Additionally, protein palmitoylation was found to be involved in reactive oxygen species regulation.

**Conclusions:**

Protein palmitoylation regulates sperm motility through calcium signaling, protein tyrosine phosphorylation, and reactive oxygen species. This study revealed the characteristics of protein palmitoylation in sperm and its role in regulating sperm motility, thereby providing novel insights into the causes of asthenozoospermia associated with sperm motility in humans.

**Supplementary Information:**

The online version contains supplementary material available at 10.1186/s40659-024-00580-4.

## Introduction

Protein palmitoylation, a crucial posttranslational modification, entails the reversible covalent attachment of a 16-carbon saturated palmitic acid to protein cysteine residues via thioester linkages [1, 2]. This modification plays a significant role in diverse biological processes, such as regulating protein stability, mediating membrane fusion, facilitating intracellular protein trafficking, and participating in cellular signaling pathways, particularly protein phosphorylation [1–3]. Protein palmitoylation is also implicated in various diseases, including cancer, neurological disorders, inflammation, metabolic disorders, infections, and neurodegenerative diseases [4, 5]. Recent studies, including ours [6], have highlighted the role of protein palmitoylation in male reproduction [7–10]. This process is dependent on the activity of the palmitoyl-acyltransferases, aspartate-histidine-histidine-cysteine (Asp-His-His-Cys, DHHC) protein family [2, 4]. The DHHC proteins and their functions have been partially profiled in testes and sperm [8, 11, 12]. Notably, protein palmitoylation in sperm may be more important than that in somatic cells because of transcriptional gene silencing in mature sperm.

Sperm motility is a critical determinant of male fertility, enabling sperm to reach and fertilize eggs. Only spermatozoa exhibiting optimal motility can traverse the cumulus cell layer and zona pellucida to fuse with the egg [13]. Motility is contingent upon various key signaling pathways and components, including proteins undergoing tyrosine phosphorylation, calcium ions (Ca^2+^) [14, 15], reactive oxygen species (ROS) [14], and heat shock protein 90 (HSP90) [16]. The protein kinase A/protein kinase C (PKA/PKC) signaling pathway plays a pivotal role in regulating protein tyrosine phosphorylation, with PKA activation leading to enhanced protein tyrosine phosphorylation and PKC promoting elevated Ca^2+^ levels [17]. These pathways are crucial for sperm function, particularly for capacitation. Ca^2+^ is vital for maintaining sperm viability, primarily through its interactions with various calcium-related proteins, its role in intracellular Ca^2+^ mobilization, and its presence in Ca^2+^ channels [18, 19]. ROS production is a double-edged sword for sperm motility; while low concentrations can enhance sperm viability, excessive levels can compromise sperm function and lead to infertility [20–22]. HSP90, a ubiquitously conserved molecular chaperone, interacts with over 250 client proteins and has been implicated in more than 300 signaling pathways [23–25]. Our previous research has established HSP90’s role in sperm motility, particularly in regulating intracellular Ca^2+^ and protein tyrosine phosphorylation, as well as its involvement in the PKA/PKC pathways [16, 26].

The location and distribution of protein palmitoylation in sperm and its regulatory role in sperm motility remain elusive, despite our previous findings linking protein palmitoylation to sperm maturity and capacitation [6]. To elucidate this, we conducted an in vitro investigation into the location and alterations of protein palmitoylation in sperm under physiological conditions, employing a novel click chemistry approach. Furthermore, we explored how protein palmitoylation influences sperm motility, focusing on signals related to PKA/PKC-associated protein tyrosine phosphorylation, Ca^2+^, ROS, and HSP90. This study revealed the physiological role of protein palmitoylation in sperm and delineated its mechanism in modulating sperm motility, which is a vital component of natural fertilization.

## Materials and methods

### Reagents and animals

The PKA inhibitor H89 dihydrochloride (H89, ab120341) and 1,2-bis-(2-aminophenoxy) ethane-N, N, N’, N’-tetraacetic acid tetra(acetoxymethyl) ester (BAPTA-AM, ab120503) were procured from Abcam (Cambridge, UK). PKC inhibitor chelerythrine chloride (CC, A10196) was sourced from AdooQ BioScience (Irvine, CA, USA). N-Ethylmaleimide (NEM, HY-D0843) and BMCC-biotin (21900) were purchased from Thermo Fisher Scientific Inc. (NC, USA). BeyoMag™ streptavidin magnetic beads (P2151), loading buffer (5×), HRP-streptavidin (A0303), bovine serum albumin (BSA, ST023), and all other reagents for sodium dodecyl sulfate-polyacrylamide gel electrophoresis (SDS-PAGE) and Western blotting were acquired from Beyotime Institute of Biotechnology (BIB, Shanghai, China). Human tubal fluid (HTF, MR-070-D) and ethylene glycol-bis (β-aminoethyl ether)-N, N, N′, N′-tetraacetic acid tetrasodium salt (EGTA, E8145) were sourced from Merck (Darmstadt, Germany). Hydroxylamine hydrochloride (NH_2_OH, 379921), dimethyl sulfoxide (DMSO, C10265), and 2-bromopalmitic acid (2BP, 2151) were procured from Sigma-Aldrich (MO, USA).

The animal study procedures were approved by the Animal Ethics Committee of the Zhejiang Academy of Medical Sciences (merged into Hangzhou Medical College). All experiments involving animals adhered to the guidelines set by the Animal Care and Use Committee of Hangzhou Medical College. Sexually mature male ICR mice, with a license for production (SCXK(Zhe) 2019–0011) and application (SYXK(Zhe) 2019-0002), aged over eight weeks, were obtained from the Experimental Animal Center of Zhejiang Province (Hangzhou, China). The animals were housed in plastic cages under specific pathogen-free conditions in a temperature-controlled room (23 ± 2 °C) with 60% ± 10% relative humidity and a 12 h light-dark cycle, as previously described [27]. Animal reporting in this study complied with the ARRIVE guidelines.

### Sperm preparation

Sperm were prepared as previously described [6]. According to experimental requirements, one–12 mice were euthanized using an overdose of CO_2_ in a manual chamber in each experiment, following the American Veterinary Medical Association Guidelines for the Euthanasia of Animals (2020). Caudal epididymal tissues were collected, minced, and incubated in pre-warmed HTF (1 mL/mouse) at 37 °C for 10 min to release the sperm. The sperm suspension was separated from the tissue and collected via gravity settling or centrifugation at 500 × g for 10 s. Sperm were harvested after centrifugation at 500 × g for 20 min, ensuring no contamination with somatic or immature spermatogenic cells. The sperm concentration was adjusted to approximately 10 × 10^6^ cells/mL using HTF, divided into aliquots, and treated with 2BP, H89, CC, BAPTA-AM, or EGTA, according to the experimental design. These aliquots were then incubated in a 5% CO_2_ incubator at 37 °C for 90 min, or the required time for the design.

Different inhibitors were used for the experimental design. 2BP, an inhibitor of protein palmitoylation [28], was used to explore whether protein palmitoylation was inhibited in mouse sperm and the effects of protein palmitoylation on sperm motility, protein tyrosine phosphorylation, and ROS. The PKA inhibitor H89 [26] and PKC inhibitor CC [29] were used to examine the effect of PKA/PKC-mediated protein phosphorylation on protein palmitoylation. Moreover, BAPTA-AM, an intracellular calcium chelator [30], and EGTA, an extracellular calcium chelator [31], were used to detect the effect of intracellular and extracellular Ca^2+^ on protein palmitoylation, respectively.

### Sperm motility analysis

Sperm motility was assessed using a computer-assisted sperm analysis (CASA) system (IVOS, Hamilton-Thorne Bio-Sciences, MA, USA) at 0 and 90 min after treatment with 25, 50, or 100 µM 2BP. The CASA parameters were set as follows [16]: frame rate, 60 Hz; acquisition frame, 45; minimum contrast, 50; minimum cell size, 5 pixels; cell intensity, 90; path velocity, 10.0 μm/s; straightness threshold, 0; slow cell, average path velocity (VAP) and straight line velocity (VSL) of less than 5.0 μm/s and 0 μm/s; temperature, 37 °C; and chamber depth, 20 μm. For analysis, 5 µL sperm samples were loaded into a pre-warmed 20 μm-depth Goldcyto chamber (SCA20-04-01; Microptic, Barcelona, Spain). Motion parameters, including VAP, VSL, straightness (STR), curvilinear velocity (VCL), linearity (LIN), amplitude of lateral head displacement (ALH), beat-cross frequency (BCF), and the percentage of motile and progressive sperm were assessed (with at least 200 sperm). Data from four replicates were collected for statistical analyses.

### Click chemistry

Click chemistry was conducted using a copper-catalyzed azide/alkyne reaction to label in situ palmitoylated proteins in sperm, following the manufacturer’s instructions. Sperm aliquots (1 mL) were cultured with or without 50 µM Click^®^ IT palmitate azide (C10265; Invitrogen, Carlsbad, CA, USA) at 37°C in a 5% CO_2_ environment for 0, 60, 90, 120, and 180 min. Sperm were washed twice with PBS and fixed with 4% paraformaldehyde at 25°C for at least 15 min. Fixed samples were smeared onto Silane-Prep Slides (S4651-72EA; Sigma Aldrich, MO, USA) and air-dried. Permeabilization was achieved using 0.25% Triton X-100 for 15 min, followed by three washes with 3% BSA in PBS. Endogenous biotin was blocked using a biotin-blocking reagent (P0101; BIB, Shanghai, China) at 25°C for 30 min, followed by three washes with 3% BSA in PBS. The samples were then incubated with the Click-IT^®^ Cell Reaction Buffer Kit (C10269; Invitrogen, Carlsbad, CA, USA) and 5 µM biotin alkyne (B10185; Invitrogen) at 25°C for 30 min, followed by three washes with 3% BSA in PBS. The samples were treated with 1 µg/mL Alexa Fluor^®^ 488 conjugated streptavidin (S11223; Invitrogen). for one hour at 25°C, followed by five washes with PBS. Nuclear staining was performed with 0.5 µg/mL 4’,6-diamidino-2-phenylindole (DAPI, D9542; Roche, Mannheim, Germany) for 5 min, followed by three washes with PBS. Observations were made under a fluorescence microscope, and images were captured using the NIS-Elements software. The mean fluorescence intensity was determined, processed by subtraction of the background, and analyzed using the ImageJ software.

### Protein extraction

The sperm samples were washed twice with PBS prior to resuspension in lysis buffer (P0013, BIB, Shanghai, China) supplemented with phenylmethylsulfonyl fluoride, cOmplete™ EDTA-free Protease Inhibitor Cocktail (11836170001; Roche, Mannheim, Germany), and phosphatase inhibitor PHOSTOP cocktail (11836170001; Roche, Mannheim, Germany). The sperm were then lysed on ice via ultrasonication at a frequency of 20 kHz, employing a cycle of 5 s, followed by 10 s off for a total duration of 30 s. Subsequently, the lysate was centrifuged at 14,000 × g at 4 °C for 30 min, and the supernatant was collected. Protein concentration in the supernatant was determined using an enhanced BCA Protein Assay Kit (P0010; BIB, Shanghai, China).

### Acyl-biotin exchange (ABE) method and streptavidin precipitation

The ABE method, as previously described [6], was employed to quantify protein palmitoylation levels. The protein lysate supernatant was precipitated using ice-cold acetone. The resulting pellets were harvested by centrifugation at 14,000 × g at 4 °C for 15 min and subsequently solubilized in cell lysis buffer (P0013; BIB, Shanghai, China) containing 50 mM NEM at 4 °C for two hours. This mixture underwent precipitation with pre-cooled acetone at − 20 °C and was split into two portions. The first portion was mixed with 150 µL of 1.5 M NH_2_OH buffer, and the second portion was mixed with 150 µL of 0.1 M Tris-HCl buffer as a control. Further precipitation was achieved using pre-cooled acetone at − 20 °C for 90 min. The pellets were treated with 0.8 µM BMCC-Biotin in Tris-HCl buffer and maintained at 4 °C for one hour. The resulting mixtures were precipitated with pre-cooled acetone at − 20 °C and re-dissolved in lysis buffer (P0013). An enhanced BCA Protein Assay Kit (P0010; BIB, Shanghai, China) was used to determine the protein concentration. The quantified protein was used for ABE experiments and streptavidin precipitation.

For the ABE experiment, the quantified protein buffer was heated in 5× loading buffer at 100 °C for 5 min. The samples were separated by SDS-PAGE and incubated with streptavidin-conjugated horseradish peroxidase (A0303; BIB, Shanghai, China). Protein bands were probed using an Enhanced Chemiluminescence Kit (Pierce Biotechnology, Rockford, IL, USA) and visualized using an Imager 600 system (Amersham™, GE Healthcare, IL, USA). When a loading control was needed, the membranes were probed using Western blotting with a β-tubulin antibody. The molecular weights of the detected proteins were compared with those of a pre-stained protein ladder (26616; Fermentas, Thermo Fisher Scientific Inc.). Gray intensity was quantified using ImageJ software.

For streptavidin precipitation, the quantified protein was incubated overnight at 4 °C with magnetic beads conjugated with streptavidin (P2151; BIB, Shanghai, China) and washed three times with PBS. The centrifuged beads, which served as protein aliquots, were subjected to Western blot analysis to detect palmitoylated HSP90.

### Western blotting analysis

This procedure was performed as previously described [26, 27]. Extracted protein aliquots were boiled in 5× loading buffer at 100 °C for 5 min. Protein aliquots were resolved using SDS-PAGE in a gel containing 12% acrylamide and transferred to polyvinylidene difluoride membranes (FFP28, BIB, Shanghai, China). The membranes were blocked with Tris-buffered saline Tween-20 (TBST; pH 7.4, 20 mM Tris-HCl, 50 mM NaCl, 0.1% Tween 20) containing 3% BSA for 2 h. After blocking, the membranes were incubated with primary antibodies against HSP90 (ab203126; Abcam, Cambridge, UK; RRID, AB_2800428; Lot, GR3359427-3; 1:1000), tyrosine phosphorylation (61-5800; Invitrogen; RRID, AB_2533927; Lot, 801118A2; 1:1000;), β-tubulin (ab6046; Abcam; RRID, AB_2210370; Lot, GR126811-1; 1:1000), and peroxidase-conjugated secondary antibodies (656120; Invitrogen; RRID, AB_2533967; Lot, VL313544; 1:10000) at 4 °C overnight, followed by three washes (5 min each) with TBST containing 0.01% (v/v) Tween-20 at 25 °C. Protein bands were detected by the SuperSignal™ West Pico PLUS chemiluminescent substrate kit (34580; Thermo Fisher Scientific Inc.), and the gray intensity was quantified using ImageJ software.

### Measurement of [Ca^2+^]_i_ level

As previously described [16], intracellular calcium (**[**Ca^2+^**]**_i_) levels in spermatozoa were measured using a fluorescent probe Fluo4-AM (S1060; BIB, Shanghai, China). Briefly, spermatozoa were loaded with 5 µL Fluo-4 AM in the dark at 37 °C in a 5% CO_2_ incubator for 30 min and then washed three times with HTF. The loaded spermatozoa aliquots were treated with 2BP or DMSO. The fluorescence intensity at 488/20 nm excitation and 516/20 nm emission wavelengths was monitored using a Cytation 3™ multifunctional microplate reader (Biotek, USA). The **[**Ca^2+^**]**_i_ levels were standardized by setting the initial recorded fluorescence intensity values as 1.00 in the blank group (the Fluo4-AM-loaded spermatozoa aliquots in HTF without any treatment) at 0 min.

### Determination of ROS levels

ROS levels were measured using the fluorescent probe 2,7-dichlorodihydrofluorescein diacetate (DCFH-DA) in ROS assay kits **(**50202ES01; Yeasen, Shanghai, China) [32]. Similar to the Ca^2+^ level detection method, the prepared sperm in HTF were loaded with 10 µL DCFH-DA for 30 min in the dark and then washed three times with HTF. Equal aliquots were treated with 2BP, DMSO, or Rosup (positive control). The fluorescence intensity at 488/20 nm excitation and 525/20 nm emission wavelengths was monitored using the same microplate reader. ROS levels were standardized as described for [Ca^2+^]_i_ measurement.

### Statistical analysis

Data are presented as mean ± standard error of the mean (SEM). Statistical analyses were conducted using the IBM SPSS Statistics version 23.0 software (IBM, USA). Initially, the normality of distribution was assessed using the Shapiro-Wilk test, and homogeneity of variance was assessed using the Levene test. If the data followed a normal distribution, one-way ANOVA, paired Student’s t-test, or Dunnett’s T3 test was employed. Alternatively, the Mann–Whitney or Wilcoxon tests were used. The threshold for statistical significance was set at *P* < 0.05.

## Results

### Location of protein palmitoylation in situ in mouse sperm

**Fig. 1:**
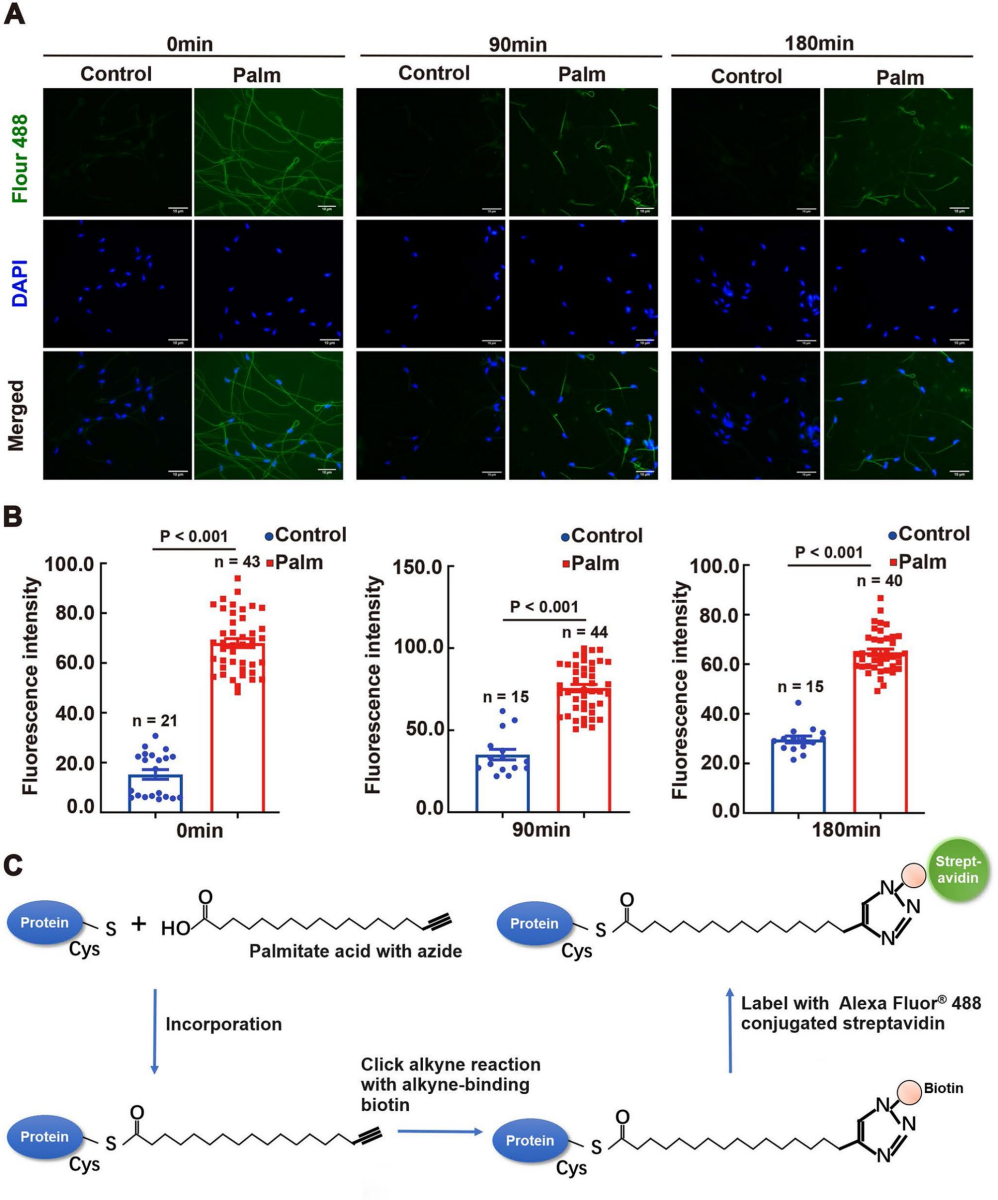
Location of Protein Palmitoylation in Mouse Sperm. (**A**) In situ protein palmitoylation in mouse sperm, with palmitoylation depicted in green (Alexa Fluor 488) and nuclei depicted in blue (DAPI). Palmitoylation (Palm) was labeled using click chemistry with azide; the negative control (Control) lacked azide. (**B**) Fluorescence intensity highlights the distinction between protein palmitoylation and the negative control. The fluorescent intensity was processed and analyzed with ImageJ. Data are presented as means ± SEM (n, number of analyzed images from three distinct samples, one mouse per individual sample). A *P*-value < 0.05 denotes statistical significance. (**C**) Schematic illustrating the procedure of the click-IT method

To observe protein palmitoylation in situ in sperm, we employed click chemistry method (schematic diagram shown in Fig. 1C). To ascertain the applicability of this method to sperm, we compared the green fluorescence intensities of Fluor^®^ 488 between the azide and azide-free control groups at 0, 90, and 180 min, respectively (Fig. 1A). A significant difference in fluorescence intensity was observed between the azide-treated group and the azide-free negative control (Fig. 1B, *P* < 0.001 for each group), indicating the successful uptake of palmitate azide by sperm and the presence of protein palmitoylation in sperm detected by click chemistry in situ.

### Distinct patterns of protein palmitoylation in situ in mouse sperm

**Fig. 2:**
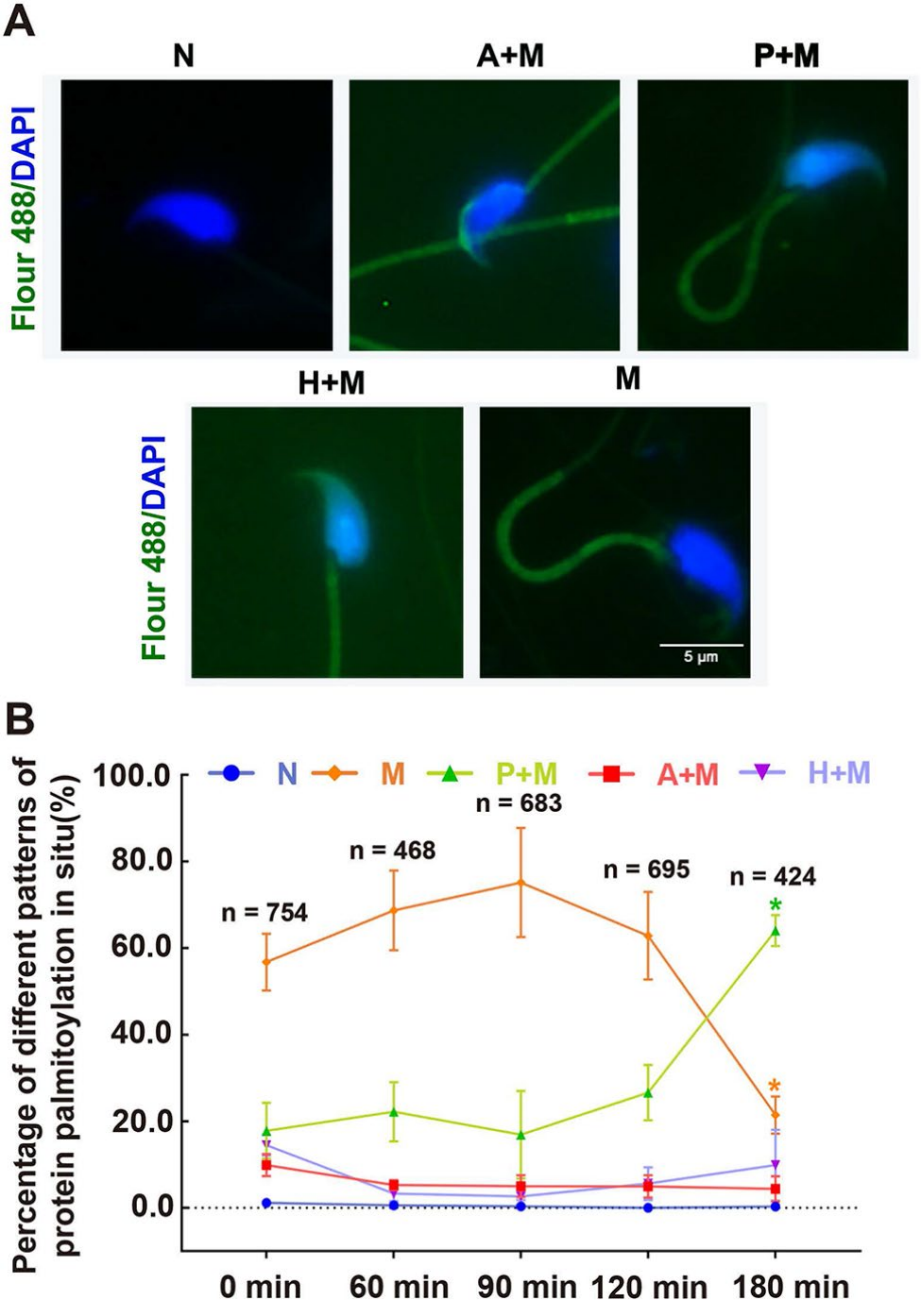
In Situ Patterns of Protein Palmitoylation in Sperm. (**A**) Different patterns of protein palmitoylation locations were observed: no fluorescence (N), acrosome and middle piece (A + M), post-acrosomal region and middle piece (P + M; more details shown in Supplementary Material 1), head and middle piece (H + M), and only the middle piece (M) exhibiting fluorescence. (**B**) Percentage of different patterns of protein palmitoylation in sperm at different time points. The proportions of different patterns of protein palmitoylation in sperm were assessed after different incubation durations using click chemistry. Data are presented as means ± SEM (n, number of analyzed sperm from three distinct samples, one mouse per individual sample)

Our results revealed the presence of protein palmitoylation in sperm incubated in HTF medium for various durations under physiological conditions in vitro. Additionally, we observed distinct patterns of protein palmitoylation in sperm (Fig. 2A), categorized as non-fluorescent (N), fluorescence with acrosome and middle piece (A + M), post-acrosomal region and middle piece (P + M; more details shown in Supplementary Material 1), head and middle piece (H + M), and middle piece (M). From 0 min to 180 min, the proportion of N ranged from (0.0 ± 0.0)% to (1.1 ± 0.8) %; P + M, from (16.9 ± 10.1) % to (64.0 ± 3.6) %; H + M, from (3.3 ± 1.8) % to (14.5 ± 2.3) %; M, from ( 21.4 ± 4.3) % to (75.1 ± 12.6)%; A + M, from (9.9 ± 2.5) % to (4.4 ± 2.9) %. Before 120 min, the proportion of M was higher than that of the other groups, whereas after 120 min, the proportion of P + M was higher than that of the other groups. These findings highlight the diverse locations of protein palmitoylation within sperm

### Changes in protein palmitoylation in sperm under physiological conditions

**Fig. 3:**
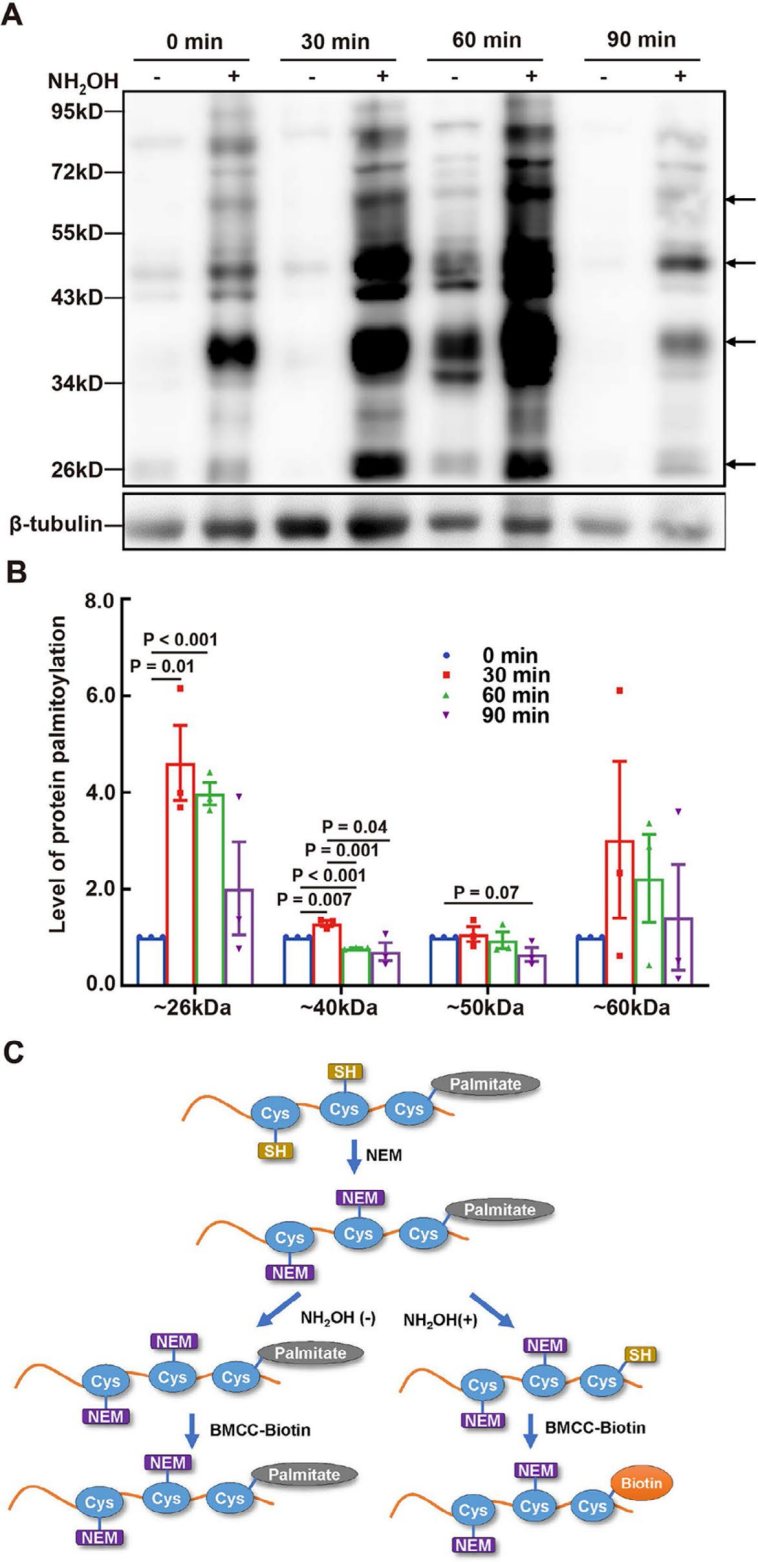
Time Course of Protein Palmitoylation Levels in Sperm. (**A**) Protein palmitoylation levels at different time points. Palmitoylation of total proteins extracted from sperm was evaluated using the ABE method with different durations of HTF incubation. The loading controls were confirmed by Western blotting. Bands in lanes with NH_2_OH represent palmitoylated proteins. (**B**) Quantification of the level of protein palmitoylation, with band gray intensities indicated by arrows, was analyzed using the ImageJ software and normalized to the total amount of loaded protein. Data are presented as means ± SEM (*n* = 3, three repeated measures from different samples). A *P*-value < 0.05 denotes statistical significance. (**C**) Schematic diagram of the ABE method. NEM, N-ethylmaleimide; NH_2_OH, Hydroxylamine; BMCC-Biotin, 1-Biotinamido-4-[4’-(maleimidomethyl)cyclohexanecarboxamido] butane

To investigate alterations in protein palmitoylation in sperm under physiological conditions, we analyzed the proportions of various patterns in situ. The results showed a notable change in the percentage of different protein palmitoylation patterns from 0 to 180 min (Fig. 2B). The main M pattern peaked at 90 min and then declined to its lowest value at 180 min. The P + M pattern was the highest at 180 min. There was a significant difference in the percentages of pattern M and P + M at 180 min compared with those at 0 min. Furthermore, we validated the protein palmitoylation level in sperm using the ABE method (schematic diagram shown in Fig. 3C), with β-tubulin serving as the loading control in Western blotting (Fig. 3A). Protein palmitoylation levels at molecular weights of approximately 26, 40, and 55 kDa, indicated by arrows, exhibited significant variations at different time points (Fig. 3B). Furthermore, it is speculated that the different molecular weights of protein palmitoylation may be limited to the post-acrosomal region and middle piece in sperm according to the proportion of patterns at different time points (Fig. 2). This suggests that protein palmitoylation in sperm changes in relation to its physiological status.

### Regulation of sperm motility by protein palmitoylation

**Fig. 4:**
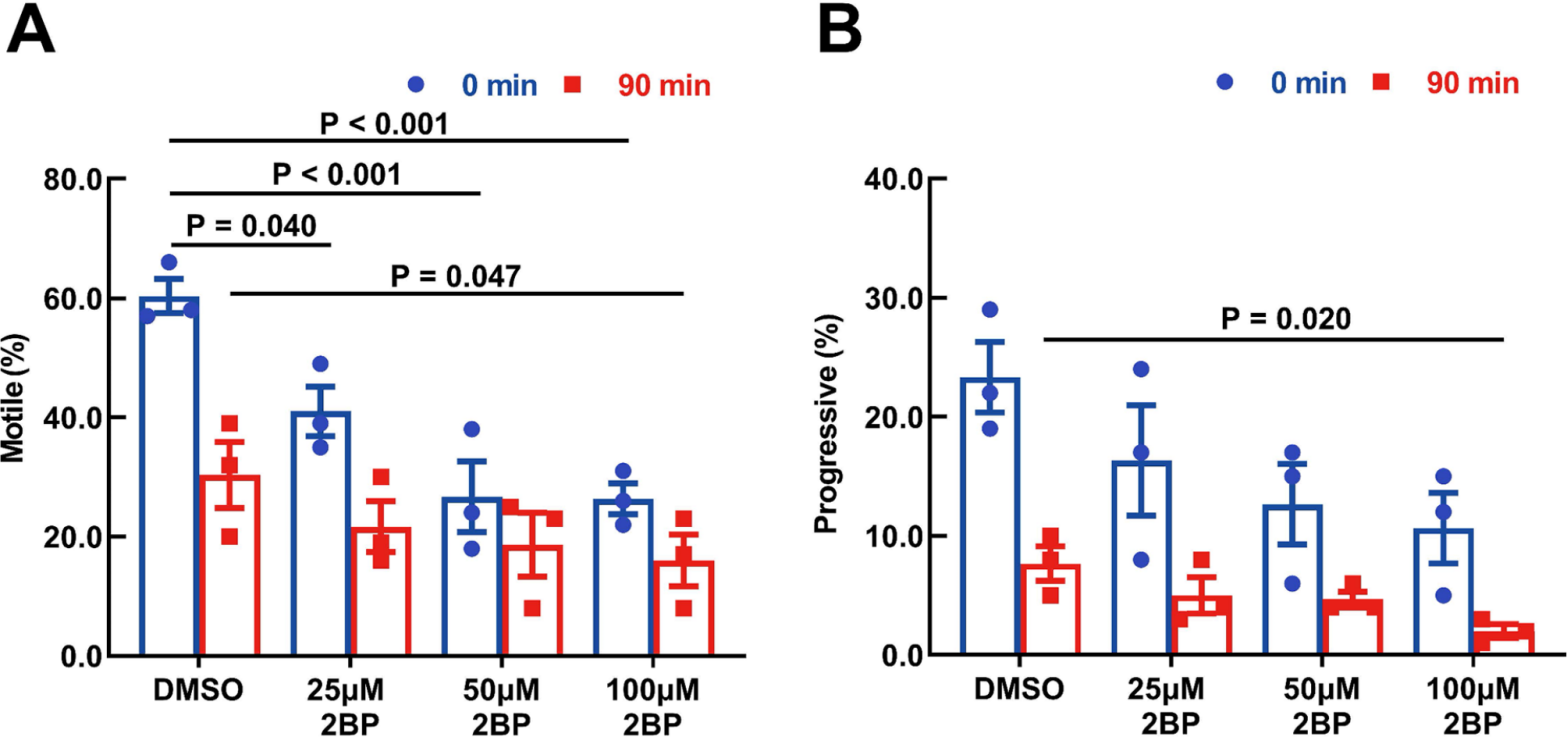
Effect of 2BP on Sperm Motility and Motion Parameters. Motion parameters, sperm motility, and progressive sperm percentage were evaluated using CASA. Data are presented as means ± SEM (*n* = 4 samples, one mouse per individual sample). The results showed that 2BP decreased the percentage of motile (**A**) and progressive sperm (**B**). A *P*-value < 0.05 denotes statistical significance

To elucidate the effects of protein palmitoylation on sperm motility, we assessed sperm motion parameters using CASA. Consistent with the findings of Goodson et al. [33], the motility profiles of the control group typically exhibited a decrease in the percentage of motile and progressive sperm over the incubation period. Our analysis revealed that 2BP significantly diminished the percentage of motile sperm at 25, 50, and 100 µM (Fig. 4A) and progressive sperm at 100 µM (Fig. 4B) compared with the DMSO control. These results suggest that protein palmitoylation plays a regulatory role in sperm motility.

### Effect of 2BP on protein palmitoylation in mouse sperm

**Fig. 5:**
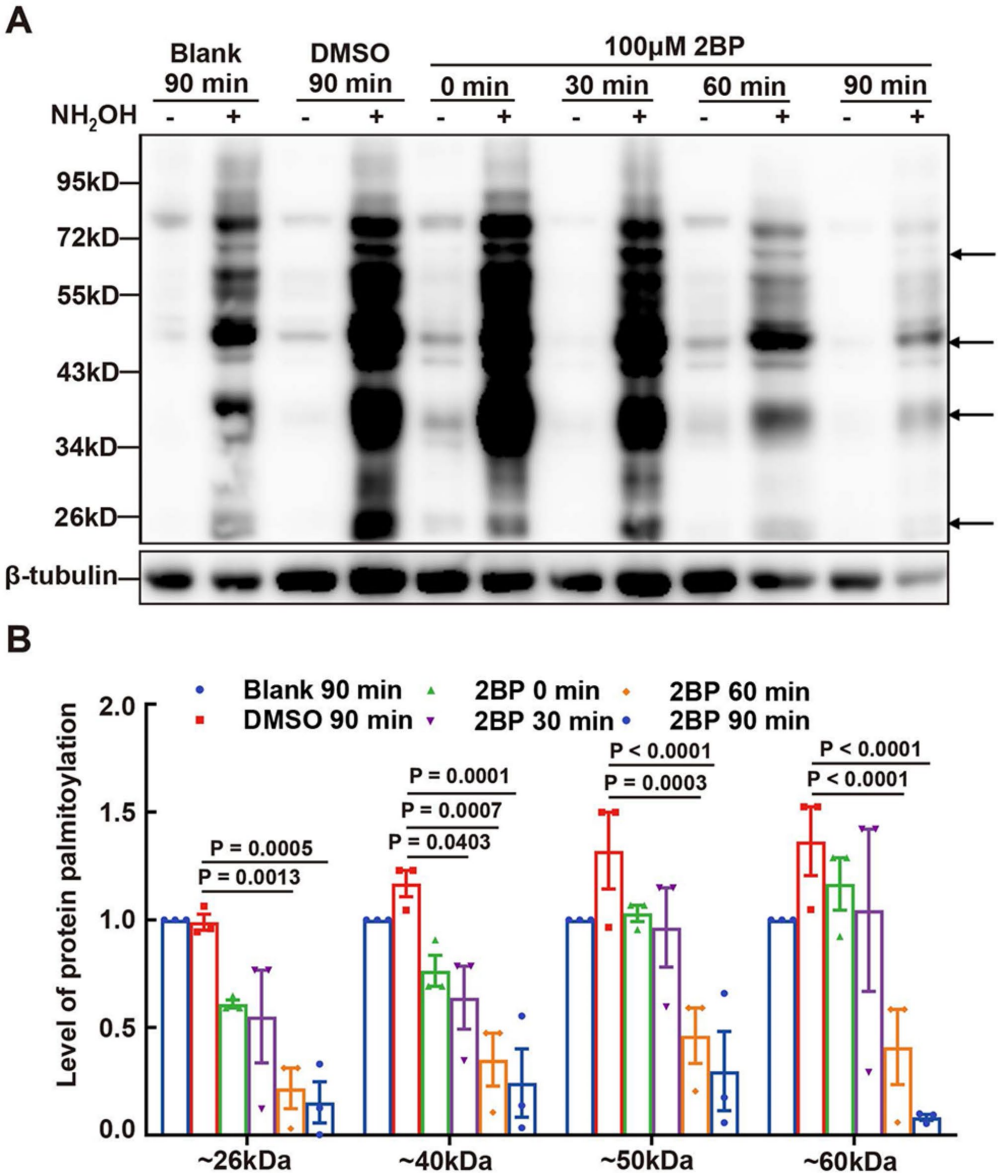
Effect of 2BP on Protein Palmitoylation in Sperm. (**A**) Effect of 2BP on protein palmitoylation in sperm. Protein palmitoylation in sperm treated with 100 µM 2BP at different time points was detected using ABE. The loading controls were confirmed by Western blotting. Bands in lanes with NH_2_OH represent palmitoylated proteins. (**B**) Quantification of protein palmitoylation levels is indicated by the arrows in (**A**). Data are presented as means ± SEM (*n* = 3 repeats, pooled samples from 12 mice per repeat), with the DMSO group as the vehicle control. A *P*-value < 0.05 denotes statistical significance

To further explore the modulation of sperm motility by palmitoylation, we investigated the effects of 100 µM 2BP, a palmitoylation inhibitor, on sperm protein palmitoylation levels. Our data indicated a decrease in protein palmitoylation levels at 30, 60, and 90 min, compared to the DMSO vehicle control at 90 min (Fig. 5A and B), although DMSO increased protein palmitoylation, compared with the blank. These findings suggest that sperm motility is regulated through protein palmitoylation, which is inhibited by 2BP.

### Interplay of protein palmitoylation with PKA/PKC-mediated protein tyrosine phosphorylation

**Fig. 6:**
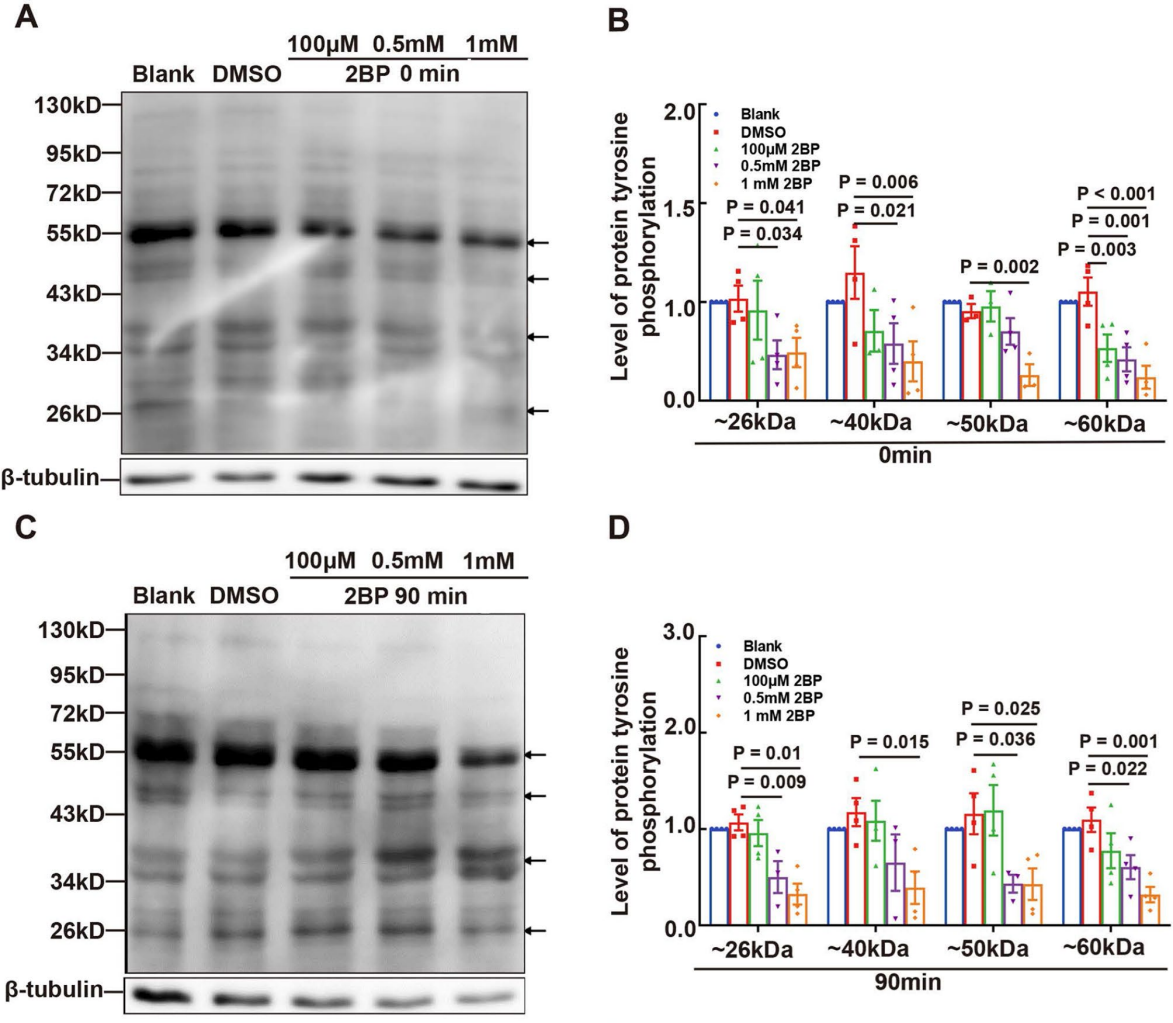
Effect of Protein Palmitoylation on Protein Tyrosine Phosphorylation in Sperm. The effect of 2BP, a protein palmitoylation inhibitor, on tyrosine phosphorylation in sperm was assessed. Protein tyrosine phosphorylation in sperm was detected by Western blotting after treatment with various concentrations of 2BP for 0 min (**A**) and 90 min (**C**), and the level of protein tyrosine phosphorylation was quantified using ImageJ software in (**B**) and (**D**), respectively; the gray intensities were normalized against β-tubulin, the loading control. Data are presented as means ± SEM (*n* = 3 or 4 repeats, pooled samples from 12 mice per repeat), with the DMSO group serving as the vehicle control. A *P*-value < 0.05 denotes statistical significance

**Fig. 7:**
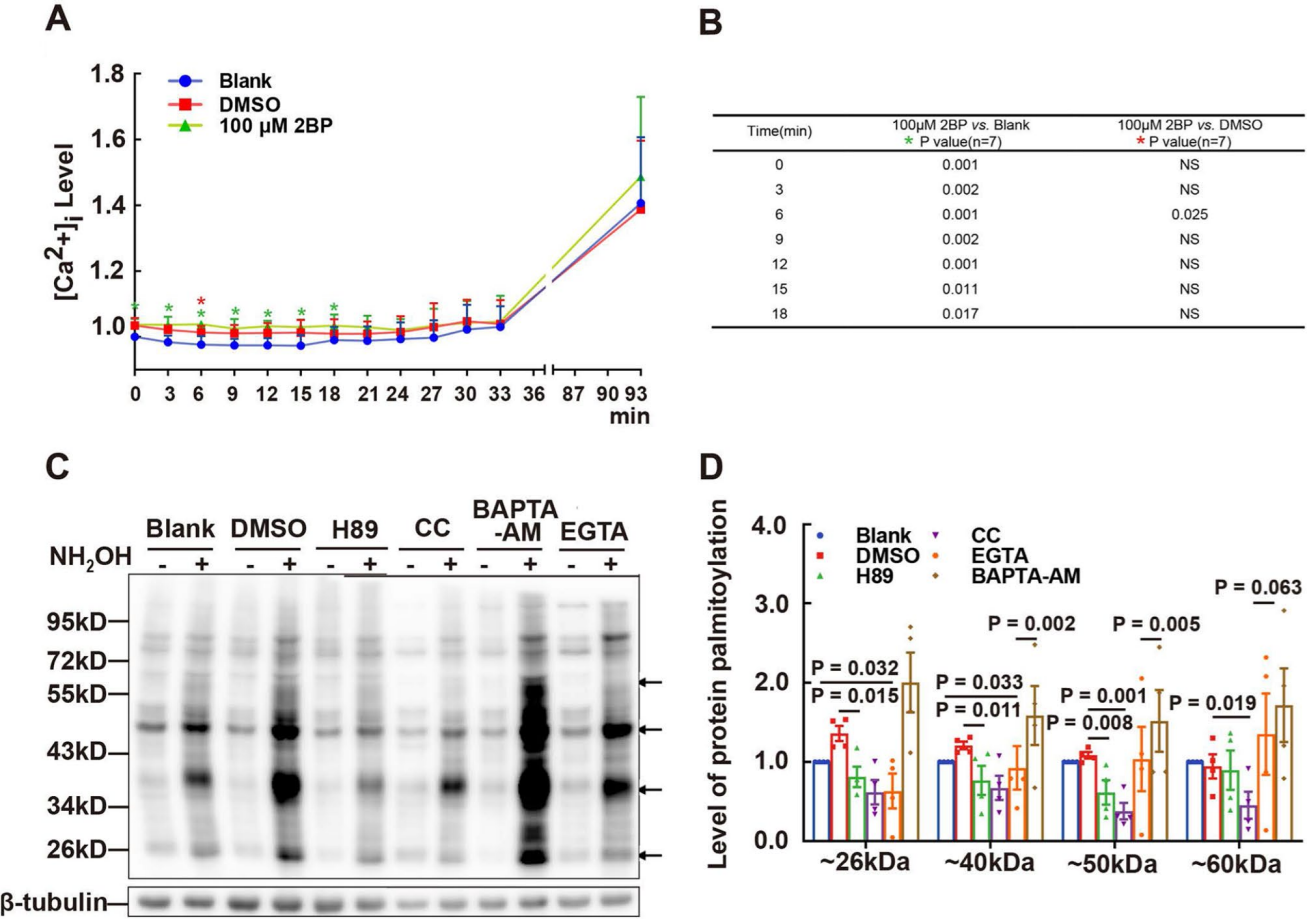
Effect of Protein Tyrosine Phosphorylation on Protein Palmitoylation and Interplay between Protein Palmitoylation and Calcium. (**A**) Effect of 2BP on intracellular calcium ([Ca^2+^]_i_) levels in sperm. Sperm loaded with the Fluo4-AM fluorescence probe were treated with 2BP, and fluorescence intensity was dynamically monitored. Data were normalized by setting initial raw intensity values to 1.00 in the blank group (*n* = 7 repeats, one individual mouse per repeat). The corresponding significant *P*-values are listed in (**B**). (**C**) Effect of PKA/PKC inhibitors and calcium chelators on protein palmitoylation in sperm. Sperm were treated with 30 µM H89, 30 µM CC, 20 µM BAPTA-AM, and 3 mM EGTA, respectively. Protein palmitoylation was detected using the ABE method. The loading controls β-tubulin were confirmed by Western blotting. The level of protein palmitoylation was quantified using ImageJ software (**D**). The gray intensities of the bands indicated by arrows in (**C**) were normalized against the total protein amount loaded. Data are presented as means ± SEM (*n* = 3 or 4 repeats, pooled samples from 12 mice per repeat), with the DMSO group serving as the vehicle control. A *P*-value < 0.05 denotes statistical significance

To investigate the relationship between protein palmitoylation and protein tyrosine phosphorylation, we examined the effects of 2BP on protein tyrosine phosphorylation and the effects of H89 and CC on protein palmitoylation. Our findings showed that 100 µM 2BP decreased the tyrosine phosphorylation levels of proteins with approximate molecular weights of 26, 40, 50, and 60 kDa at 0 (Fig. 6A and B) and 90 min (Fig. 6C and D). Similarly, H89 reduced the palmitoylation levels of proteins at approximately 40 and 50 kDa (Fig. 7C and D), whereas CC decreased the palmitoylation levels of proteins near 26, 50, and 60 kDa (Fig. 7C and D). These results suggest a complex interplay between protein palmitoylation and protein tyrosine phosphorylation in sperm, which is implicated in the regulation of sperm motility.

### Interplay of protein palmitoylation with intracellular calcium

To explore the relationship between protein palmitoylation and calcium signaling in sperm, we assessed the effects of 2BP on protein palmitoylation and the influence of BAPTA-AM and EGTA on protein palmitoylation. Our data indicated that 2BP caused fluctuations in **[**Ca^2+^**]**_i_ levels in sperm, particularly during the initial 18 min (Fig. 7A and B). Additionally, BAPTA-AM increased the palmitoylation levels of proteins with approximate molecular weights of 40, 50, and 60 kDa, whereas EGTA altered the palmitoylation levels of proteins near 26 and 40 kDa (Fig. 7C and D). These findings suggest a connection between protein palmitoylation and intracellular calcium signaling in sperm, with implications for the regulation of sperm motility.

### Regulation of HSP90 palmitoylation by PKA/PKC and calcium ions

**Fig. 8:**
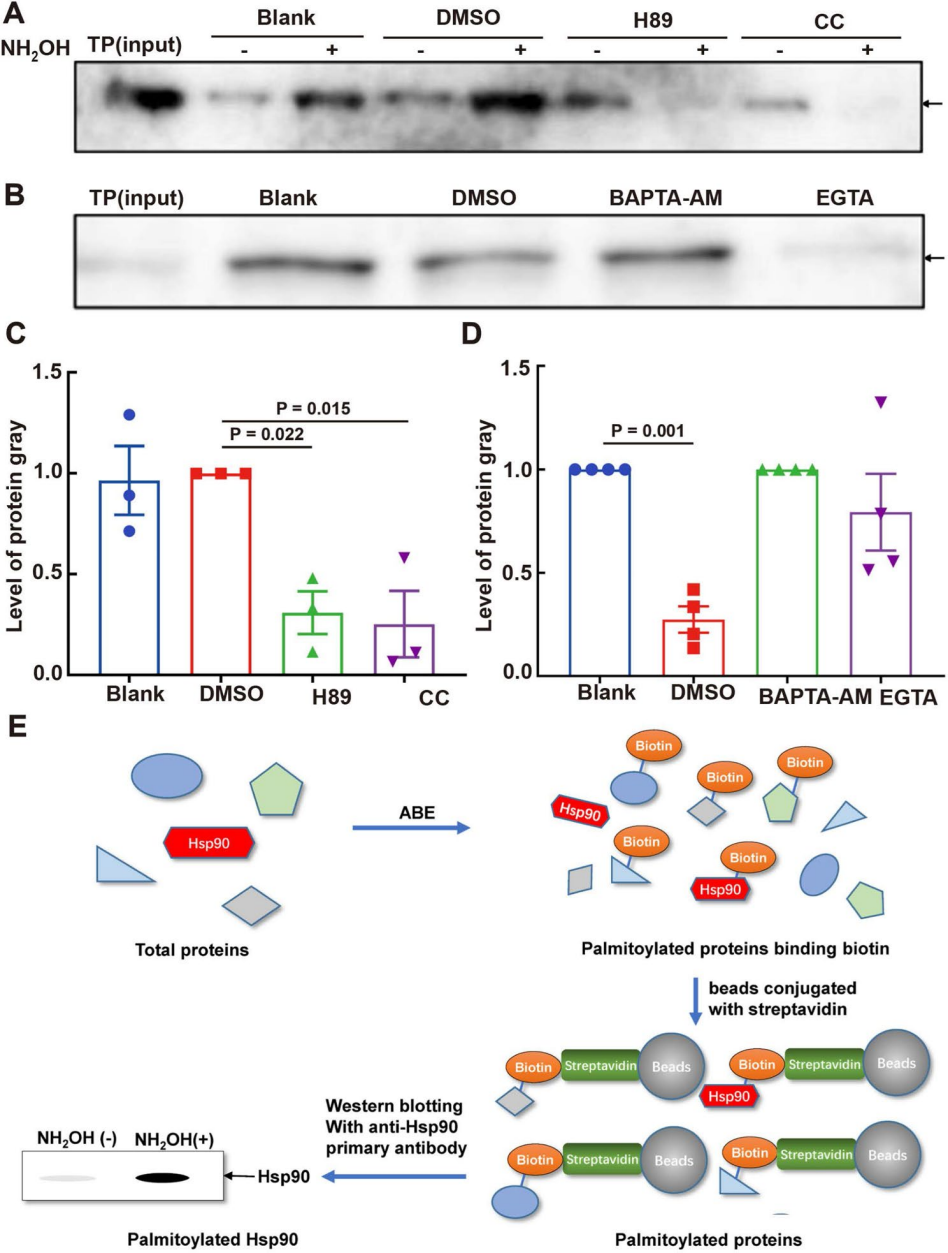
Regulation of Palmitoylated HSP90 by PKA/PKC Inhibitors and Calcium Chelators. (**A**) Effect of H89 and CC on palmitoylated HSP90. The bands indicated by the arrow are palmitoylated HSP90 with NH_2_OH, with quantification shown in (**C**). (**B**) Effect of calcium chelators on palmitoylated HSP90, with quantification displayed in (**D**). (**E**) Procedures for detecting palmitoylated HSP90: palmitoylated HSP90 was detected using an anti-HSP90 primary antibody after streptavidin-conjugated beads bound to biotinylated proteins were treated using the ABE method and separated using SDS-PAGE. Gray intensities of palmitoylated HSP90 were quantified using ImageJ software and normalized to the total amount of loaded protein. Data are presented as means ± SEM (*n* = 3 or 4 repeats, pooled samples from 12 mice per repeat), with the DMSO group serving as the vehicle control. A *P*-value < 0.05 denotes statistical significance

To further investigate the modulation of protein palmitoylation by protein tyrosine phosphorylation and calcium, we focused on HSP90 as a representative protein. Our analysis revealed that the PKA inhibitor H89, PKC inhibitor CC (Fig. 8A and C), and EGTA (Fig. 8B and D) reduced HSP90 palmitoylation. The procedure for detecting HSP90 palmitoylation is outlined in Fig. 8E. These results confirmed the involvement of protein palmitoylation in PKA/PKC-mediated protein tyrosine phosphorylation and calcium ion signaling in sperm, with implications for sperm motility regulation.

### Association of protein palmitoylation with ROS

**Fig. 9:**
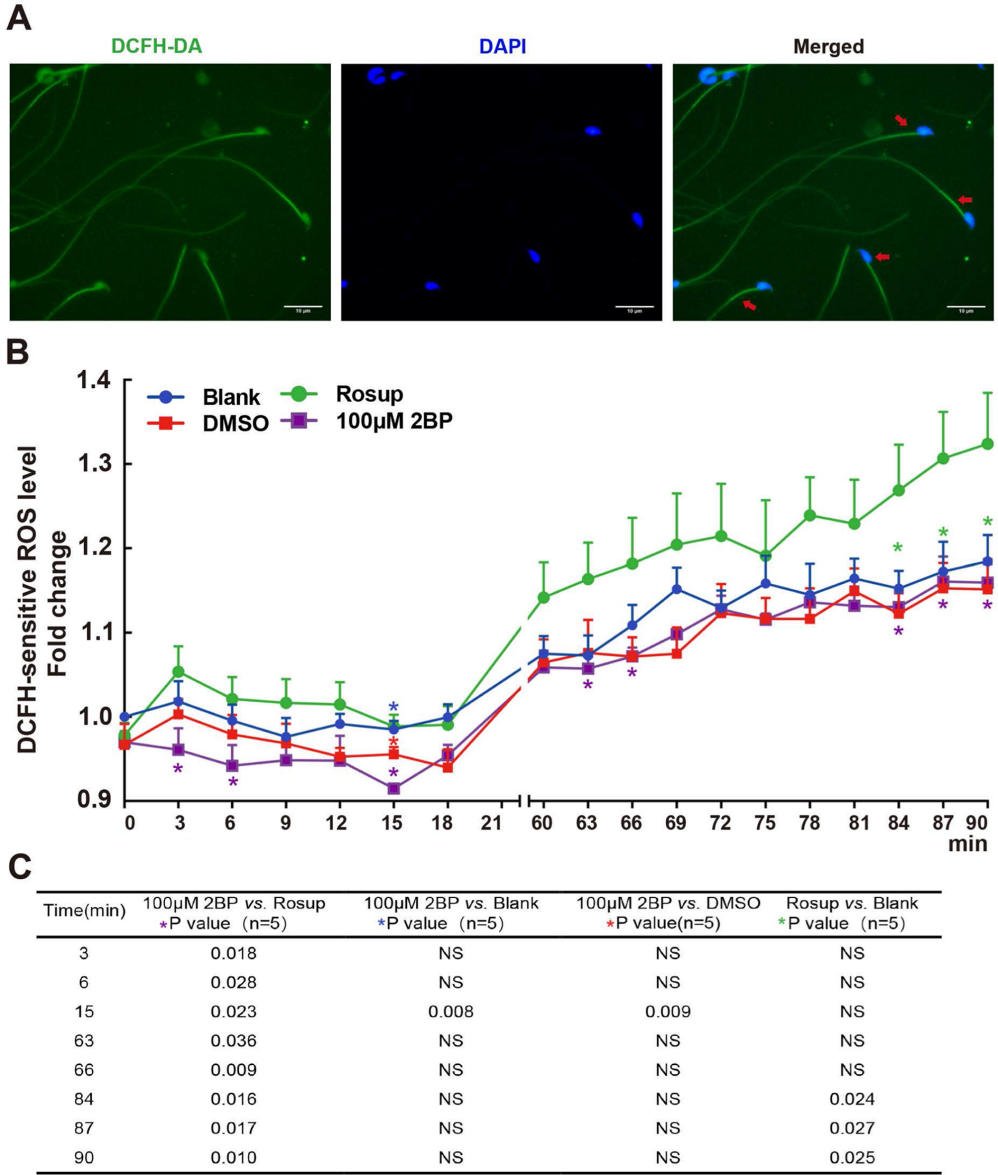
Association between Protein Palmitoylation and ROS Levels in Sperm. (**A**) DCFH-DA probes indicating ROS signals were observed in sperm. (**B**) Effect of 2BP on ROS levels in sperm. Sperm loaded with the DCFH-DA probe were treated with 2BP, and fluorescence intensity was dynamically monitored. The data were standardized by setting initial raw intensity values to 1.00 in the blank group. Data are presented as means ± SEM (*n* = 5 repeats, one individual mouse per repeat). A *P*-value < 0.05 denotes statistical significance. Significant *P*-values between different comparisons at the corresponding times are shown in (**C**)

To explore the potential correlation between protein palmitoylation and ROS, we assessed ROS levels in sperm treated with 2BP. Figure 9A shows a fluorescence indicative of ROS location in sperm. Our analysis revealed that 100 µM 2BP decreased the ROS levels in sperm from 0 to 18 min, followed by an increase (Fig. 9B). The corresponding *P*-values are shown in Fig. 9C. These findings suggest a potential association between ROS and protein palmitoylation in sperm.

## Discussion

To our knowledge, this is the first study to delineate the spatial distribution of protein palmitoylation and its associated physiological changes in sperm using in situ click chemistry. The distribution of palmitoylated proteins in various regions suggests a widespread role of this posttranslational modification in sperm function. Dynamic changes in protein palmitoylation levels, corresponding to changes in physiological status in vitro, indicate a regulatory role in sperm function. These observations suggest a regulatory role of protein palmitoylation in sperm physiology, consistent with our previous work reporting numerous palmitoylated proteins in sperm [6]. Moreover, our findings align with those of previous reports highlighting the dynamic nature of protein palmitoylation [34], suggesting that it may serve as a rapid and reversible switch for protein function in sperm. This is particularly significant given the transcriptionally and translationally quiescent nature of mature sperm. This study also validates the application of click chemistry as a powerful tool for investigating real-time physiological processes [35].

This study highlights that in sperm, protein palmitoylation is crucial for signaling pathways. Recent studies have identified over 600 palmitoylated proteins in the mammalian brain [36] and numerous palmitoylated proteins across various cell types in testicular tissues [8]. Crucially, this study has extended the findings from other tissues to mature sperm, providing a foundation for understanding the specific roles of protein palmitoylation in male fertility. In somatic and cancer models, protein palmitoylation influences downstream events, including cell proliferation, survival, invasion, metastasis, and antitumor immunity [37]. Similar to other cell models, protein palmitoylation in sperm may affect downstream events of sperm functions, such as motility, hyperactivation, capacitation, and the acrosome reaction. Therefore, these findings may have broader implications for understanding protein palmitoylation in other contexts, potentially impacting fields such as cancer research and developmental biology.

This study underscores the pivotal role of protein palmitoylation in regulating sperm motility. Our results indicate that 2BP reduces the percentage of motile and progressive sperm. Moreover, our data suggest that 100 µM 2BP most significantly reduced protein palmitoylation levels (Fig. 5), more so than higher concentrations of 500 µM or 1000 µM 2BP (data not shown), implying that the inhibitory effect of 2BP on protein palmitoylation may not follow a dose-dependent pattern. This observation aligns with findings in rat myocytes, where 100 µM 2BP nearly completely inhibited the cells, whereas 500 µM resulted in only a 10% reduction [38]. This phenomenon may be explained by several factors: 2BP may affect cellular mechanisms beyond palmitoylation, such as the depalmitoylation activity of acyl-protein thioesterases [39], the depalmitoylation process itself [40], and senescence markers [41], potentially causing unintended effects, and sperm respond uniquely to 2BP compared to other cell types. Furthermore, the impact of 2BP appeared to be time-dependent, with protein palmitoylation levels decreasing progressively, similar to the temporal dynamics of 2BP inhibition observed in another study [42]. These findings underscore the regulatory role of protein palmitoylation, modulated by 2BP, in sperm motility, in agreement with previous research demonstrating the influence of 2BP on the gliding motility of tachyzoites [43]. These results contribute to our understanding of how sperm movement is controlled by protein palmitoylation at the molecular level.

Protein palmitoylation is crucial for regulating diverse somatic cellular motilities. The palmitoylation of DHHC5 [44], claudin-7 [45], HGAL [46], and tubulin [47] is involved in stimulation-dependent plasma membrane motility, overall cell motility, chemoattractant-induced cell motility, and flagellar formation and motility, respectively. Palmitoylation and myristoylation target molecular signaling proteins such as Lck, Fyn, and Yes-1 towards motile flagella [48], potentially influencing sperm motility. Recent yeast research has demonstrated that palmitoylation may alter the functionality of yeast vac8, a homolog of ARMC3 in sperm, which is essential for sperm motility [49]. Furthermore, flagellar dynamics can be linked to tubulin palmitoylation [50]. According to the results of this study, tubulin was close to ~ 50 kDa of palmitoylated proteins; Lck, Yes, and Fyn, ~ 60 kDa; and HGAL and Claudin-7, ~ 26 kDa. Consequently, further identification of palmitoylated proteins is required, and future studies should seek direct evidence to substantiate the causal relationship between palmitoylation of specific proteins and sperm motility.

The role of protein palmitoylation in protein tyrosine phosphorylation in sperm remains to be established, although it is well known that PKA/PKC-mediated protein tyrosine phosphorylation regulates sperm motility and hyperactivation, a distinct pattern from motility, during capacitation [51–54]. Our findings revealed that varying concentrations of 2BP attenuated the tyrosine phosphorylation of specific proteins in sperm. This result suggests that protein palmitoylation may regulate protein tyrosine phosphorylation in sperm. The low toxicity of 2BP is evident, according to a report in the literature, that acute promyelocytic leukemia cells remained viable even when treated with concentrations as high as 5 or 10 mM [55]. In contrast, H89, a PKA-specific inhibitor, and CC, a PKC-specific inhibitor, partially inhibited protein palmitoylation. H89 decreased the motion parameters VAP and VCL. In contrast, CC diminished the percentage of motile sperm and the percentages of progressive sperm, VAP, VSL, and ALH (Supplementary Material 2). These results imply that sperm motility is regulated by the interplay between protein palmitoylation and PKA/PKC-mediated tyrosine phosphorylation.

Our findings are consistent with a previous report indicating that the PKA/PKC-mediated protein tyrosine phosphorylation signaling pathway influences sperm motility [51]. Furthermore, our observations demonstrated an association between protein palmitoylation and phosphorylation in sperm. Similar reciprocal regulation has been documented for neuronal proteins, including synapsin 1 via PKA regulation [56], dopamine transporter proteins [57], and GAP43 [58] via PKC regulation. These studies affirm the crucial role of PKA and PKC in the association between palmitoylation and phosphorylation. Beyond signal transduction, the physiological significance of bidirectional regulation between dynamic protein palmitoylation and phosphorylation may lie in determining protein shuttling among intracellular compartments [58]. Additionally, palmitoylation interacts with protein kinases such as Src family kinases [59], AKT [60], and PKC [61], where it is essential for the localization and function of Src family kinases [62] and PKC’s membrane translocation [61]. Conversely, PKC, in turn, regulates palmitoylation [63]. Taken together, our results suggest that protein palmitoylation governs sperm motility via its association with PKA/PKC-mediated protein tyrosine phosphorylation. The reciprocal relationship observed between palmitoylation and PKA/PKC-mediated phosphorylation aligns with previous findings on neuronal proteins, indicating a conserved regulatory mechanism across different cell types. This bidirectional regulation may serve as a fine-tuning mechanism for protein localization and function within sperm, potentially influencing their ability to respond to environmental cues during fertilization.

How protein palmitoylation interacts with calcium signaling within sperm remains uncertain, despite numerous studies highlighting the importance of Ca^2+^ homeostasis in the regulation of sperm motility. Contrary to protein tyrosine phosphorylation, our study demonstrated that protein palmitoylation controls intercellular calcium levels in sperm, with 2BP inducing elevation in **[**Ca^2+^**]**_i_ levels. Moreover, BAPTA-AM and EGTA modified the levels of partially palmitoylated proteins. Both EGTA and BAPTA-AM decreased the percentage of progressive sperm, VAP, VSL, and ALH. Additionally, BAPTA-AM led to a reduction in the percentage of motile sperm (Supplementary Material 3). Our findings align with those of a previous study, which reported an increase in intracellular calcium due to protein palmitoylation in somatic cells [64]. A potential explanation for this increased calcium influx could be that palmitoylation impedes sodium-calcium exchanger 1 (NCX1) by blocking calcium efflux and preventing desensitization of the purinergic P2 X7 receptor (P2 X7) [64], as both NCX1 and P2 X7 are implicated in the regulation of sperm motility [65, 66]. This result indicated the involvement of palmitoylation in sperm motility. In addition, Ca^2+^ appears to regulate protein palmitoylation in the sperm, which may be attributed to the upstream PKC activation of calcium channels to release calcium ions or downstream protein tyrosine phosphorylation from the PKA signaling pathway, a soluble adenylyl cyclase (sAC)-cAMP-PKA signaling pathway, as Ca^2+^ is known to activate sAC [18]. While 2BP raises **[**Ca^2+^**]**_i_ levels, it diminishes sperm motility, which could be attributed to sperm motility relying on the intricate cooperation of various factors, including **[**Ca^2+^**]**_i_ levels and protein tyrosine phosphorylation [14, 15]. Furthermore, palmitoylation modulates the functions of many Ca^2+^ transporters, affecting Ca^2+^ signaling and fluxes, including voltage-gated Ca^2+^ channels, CaV1.2, CALHM1, and CALHM13; cation channels, TRPC1, TRPC5, TRPM6, TRPM7, TRPM8, and TRPP3; Ca^2+^ release channels, TRPML1 and TRPML3; and store-operated Ca^2+^ channel, ORAI1 [67]. In summary, these findings suggest an interplay between palmitoylated proteins and intracellular calcium levels in regulating sperm motility. This study provides valuable insights into the complex interplay between protein palmitoylation and calcium signaling in sperm, revealing a previously underappreciated mechanism for regulating sperm motility. These findings challenge the conventional focus on protein tyrosine phosphorylation and highlight the critical role of protein palmitoylation in the control of intracellular calcium levels.

Our research indicates that protein palmitoylation includes palmitoylation of HSP90, a representative protein. H89, CC, and EGTA altered HSP90 palmitoylation, whereas 2BP modified the protein palmitoylation and HSP90. These results agree with those of our previous study, which reported the presence of palmitoylated proteins in sperm and demonstrated the involvement of HSP90 palmitoylation in sperm maturation and capacitation. The study noted higher levels of HSP90 palmitoylation in the cauda epididymis than in the caput epididymis and an increase in palmitoylation levels post-capacitation [6]. Furthermore, our prior research established that PKA/PKC regulates HSP90 tyrosine phosphorylation and that HSP90 plays a role in calcium regulation and control of sperm motility [16]. Moreover, protein palmitoylation interacts with heat shock proteins, including HSP20 [68], HSP27 [69], and HSP90 [6, 70], affecting their functionality. For instance, N-terminal palmitoylation of Toxoplasma gondii HSP20 is necessary for inner membrane complex localization [68], and HSP90α palmitoylation facilitates androgen to estrogen conversion [70]. Palmitoylation also influences proteins associated with heat shock proteins, such as estrogen receptor alpha (ERα) bound by HSP27, which requires palmitoylation for membrane localization and function [69]. These results further imply that HSP90 palmitoylation interacts with PKA/PKC-mediated protein tyrosine phosphorylation and calcium signaling, given that the regulatory signals for sperm motility coincide with sperm capacitation [16, 26]. The present study provides a foundation for future research to explore the precise mechanisms by which HSP90 palmitoylation influences sperm physiology and to investigate potential therapeutic interventions based on modulating this process.

In addition, 2BP influenced ROS production. Elevated ROS levels can impede sperm motility, damage mitochondria, reduce energy availability, and cause DNA damage, ultimately reducing the number of sperm capable of reaching the oocyte for fertilization [22]. Protein palmitoylation might affect ROS production via proteins containing cysteine sulfur, which participates in thiol oxidation from –SH to SS- [21], potentially disrupting ROS balance. Our study revealed that 2BP temporarily decreased ROS levels for 15 min, after which the ROS levels in various groups increased, indicating dynamic regulation. Furthermore, the modulation of ROS through protein palmitoylation could be related to tyrosine nitration and S-glutathionylation [21]. Alterations in thiol oxidation, tyrosine nitration, and S-glutathionylation, which are associated with ROS production during protein palmitoylation, influence sperm motility [21]. Therefore, it seems plausible that ROS plays a role in increasing protein palmitoylation and regulating sperm motility. This finding implies that manipulating protein palmitoylation could potentially be used as a strategy to modulate ROS levels and consequently influence sperm motility. These insights could lead to the development of novel approaches to enhance sperm motility.

Although this study explored several possible mechanisms, numerous questions remain for future research. Among these, the potentially critical role of palmitoylation in sperm function beyond motility, such as fertilization, should be studied further. Additionally, the regulatory mechanism linking protein palmitoylation and calcium homeostasis should be investigated, considering the complexity of calcium regulation, including various calcium channels, exchangers, and calcium-ATPases in sperm [15, 18, 71, 72]. Which palmitoyl-acyltransferases predominantly contribute to protein palmitoylation in sperm? How does intricate signal crosstalk occur between protein palmitoylation and phosphorylation in sperm? What implications do protein palmitoylation have for male infertility and asthenozoospermia?

**Fig. 10:**
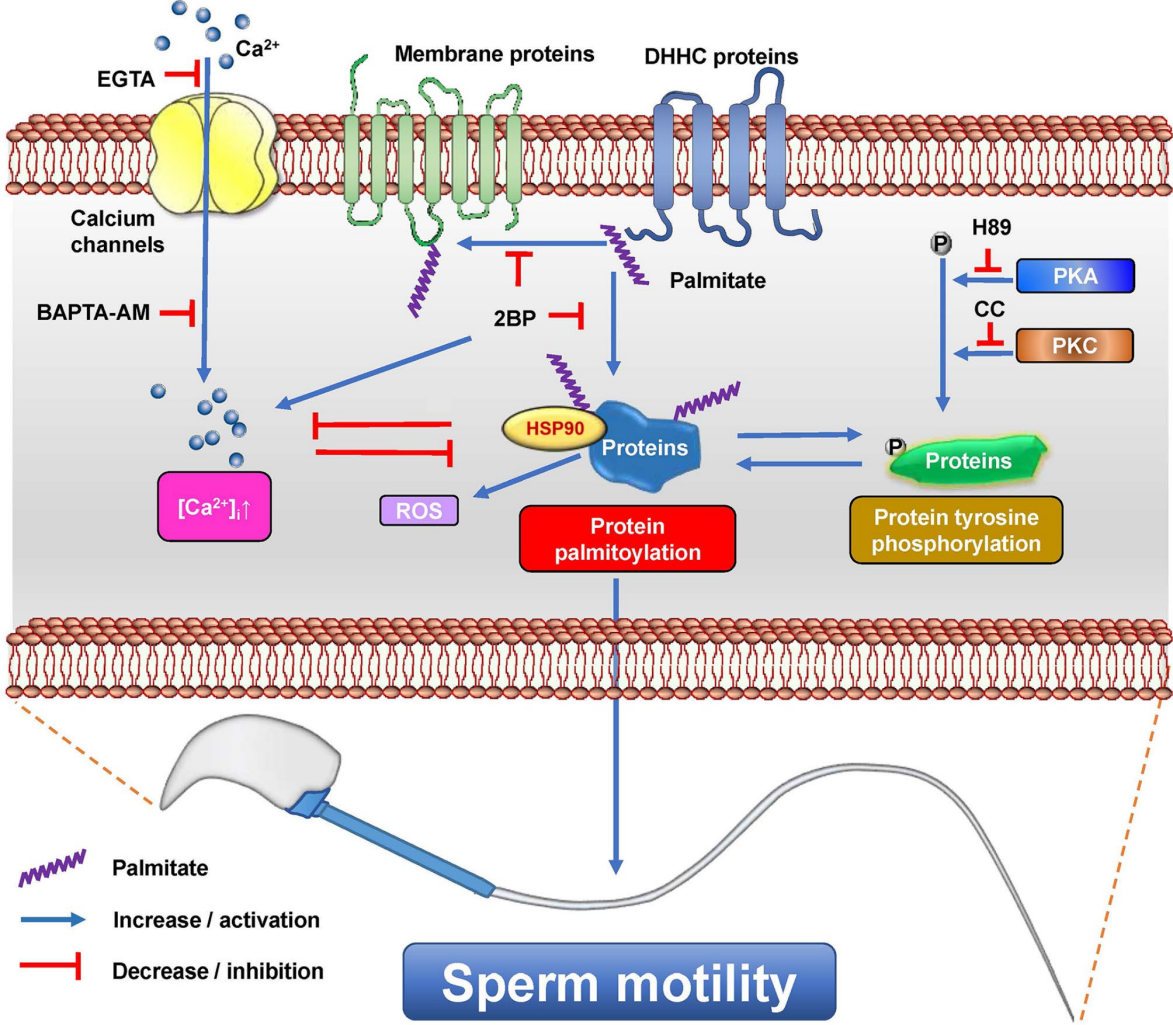
Schematic representation of the involvement of protein palmitoylation in sperm motility

In conclusion, this study identified the in situ locations of protein palmitoylation in sperm, demonstrated the dynamics of protein palmitoylation in response to physiological changes, and elucidated the crucial role of protein palmitoylation in sperm motility and a possible mechanism by coordinating PKA/PKC-mediated protein tyrosine phosphorylation, calcium homeostasis, and ROS (Fig. 10). This study has uncovered the physiological characteristics of protein palmitoylation in sperm and provided new insights into the regulatory mechanism of sperm motility, providing a potential clue to the causes of asthenozoospermia associated with sperm motility in humans. This study suggests several possible clinical translational applications for future research. First, diagnostic tools may be developed based on the correlation among palmitoylated protein levels, distribution patterns of protein palmitoylation, and sperm motility or male fertility. Second, assisted reproductive technology may be optimized by integrating palmitoylation assessment into sperm selection for in vitro fertilization and intracytoplasmic sperm injection and by developing culture media or conditions that maintain or enhance sperm palmitoylation. Third, drug therapy strategies targeting protein palmitoylation in sperm should be explored to improve motility in asthenozoospermia patients in the clinical context.

## Electronic supplementary material

Below is the link to the electronic supplementary material.

Supplementary Material 1

Supplementary Material 2

Supplementary Material 3

## Data Availability

The dataset supporting the conclusions of this study is included in the article and its Supplementary Materials.
