# Supplementary material for: Protein palmitoylation is involved in regulating mouse sperm motility via the signals of calcium, protein tyrosine phosphorylation and reactive oxygen species
Source: Biol Res. 2025 Jan 15;58:3. doi: 10.1186/s40659-024-00580-4 (PMC11734517; doi:10.1186/s40659-024-00580-4)

Additional File 1.

Location details of the post-acrosomal region and middle piece pattern

To observe the location details of the post-acrosomal region and middle piece (P+M) pattern being difficult to identify, three representative sperms, including the separated and merged colors, are shown. The top P+M 1 is the same as the P+M in Figure 2A, the middle, the different P+M 2, and the right of the bottom, the other P+M 3, left, head and middle piece (H+M).

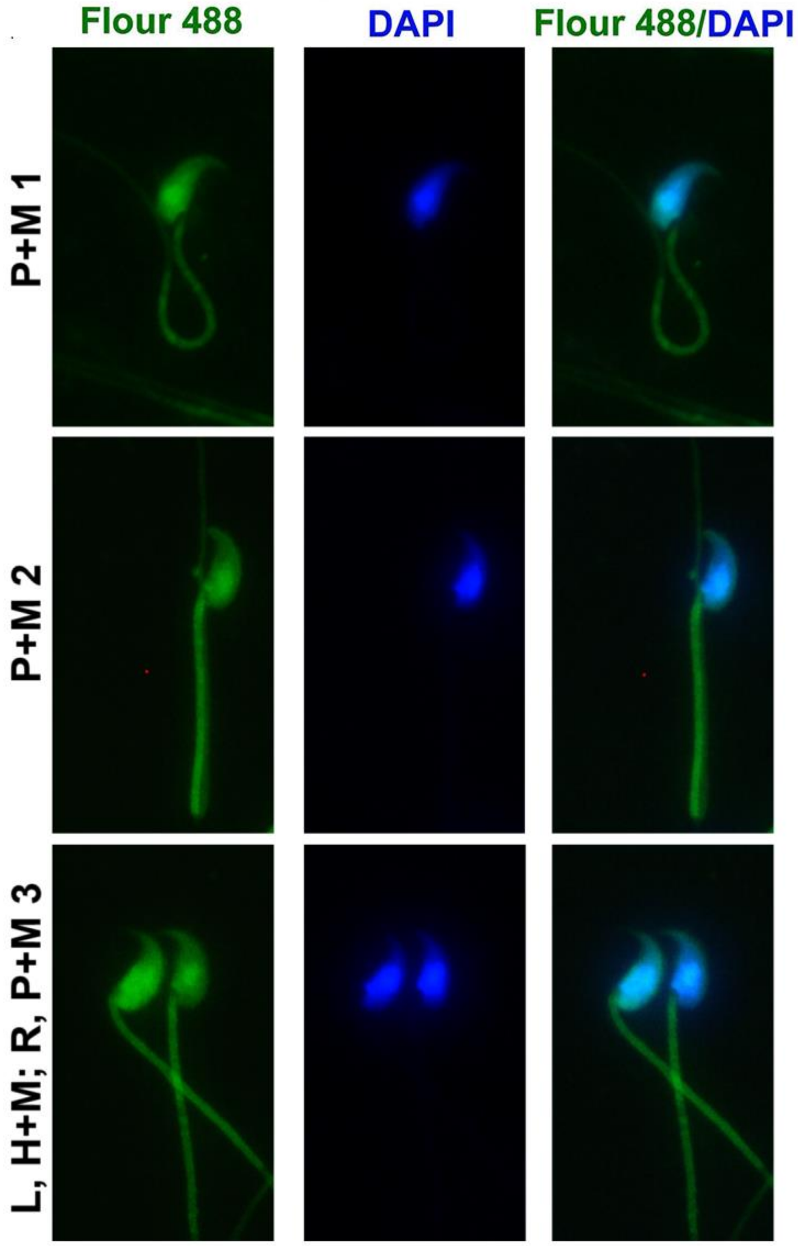

Supplement: Supplementary file 1 — Supplementary Material 1 [file 40659_2024_580_MOESM1_ESM.pdf]
