# Supplementary material for: Protein palmitoylation is involved in regulating mouse sperm motility via the signals of calcium, protein tyrosine phosphorylation and reactive oxygen species
Source: Biol Res. 2025 Jan 15;58:3. doi: 10.1186/s40659-024-00580-4 (PMC11734517; doi:10.1186/s40659-024-00580-4)

Additional File 2.

PKA and PKC regulated sperm motility and motion parameters

To explore whether PKA and PKC regulate sperm motility, we carried out the CASA analysis of sperm after sperm were treated with 30μM H89, PKA inhibitor, and 10μM CC, PKC inhibitor. The results showed H89 decreased VAP (Figure C), VCL (Figure E), and CC decreased the percentage of motile sperm (Figure A), the percentage of progressive sperm (Figure B), VAP (Figure C), VSL (Figure D), and ALH (Figure E). The results suggest that PKA and PKC regulate sperm motility, respectively. VAP, Average path velocity; VSL, straight-line velocity; VCL, curvilinear velocity; STR, straightness (STR = VSL/VAP × 100); LIN, linearity (LIN = VSL/VCL × 100); ALH, the amplitude of lateral head displacement; BCF: beat-cross frequency. The data are presented as means ± SEMs(n=5). DMSO group, as vehicle control; $P < 0.05$, set as a statistical significance.

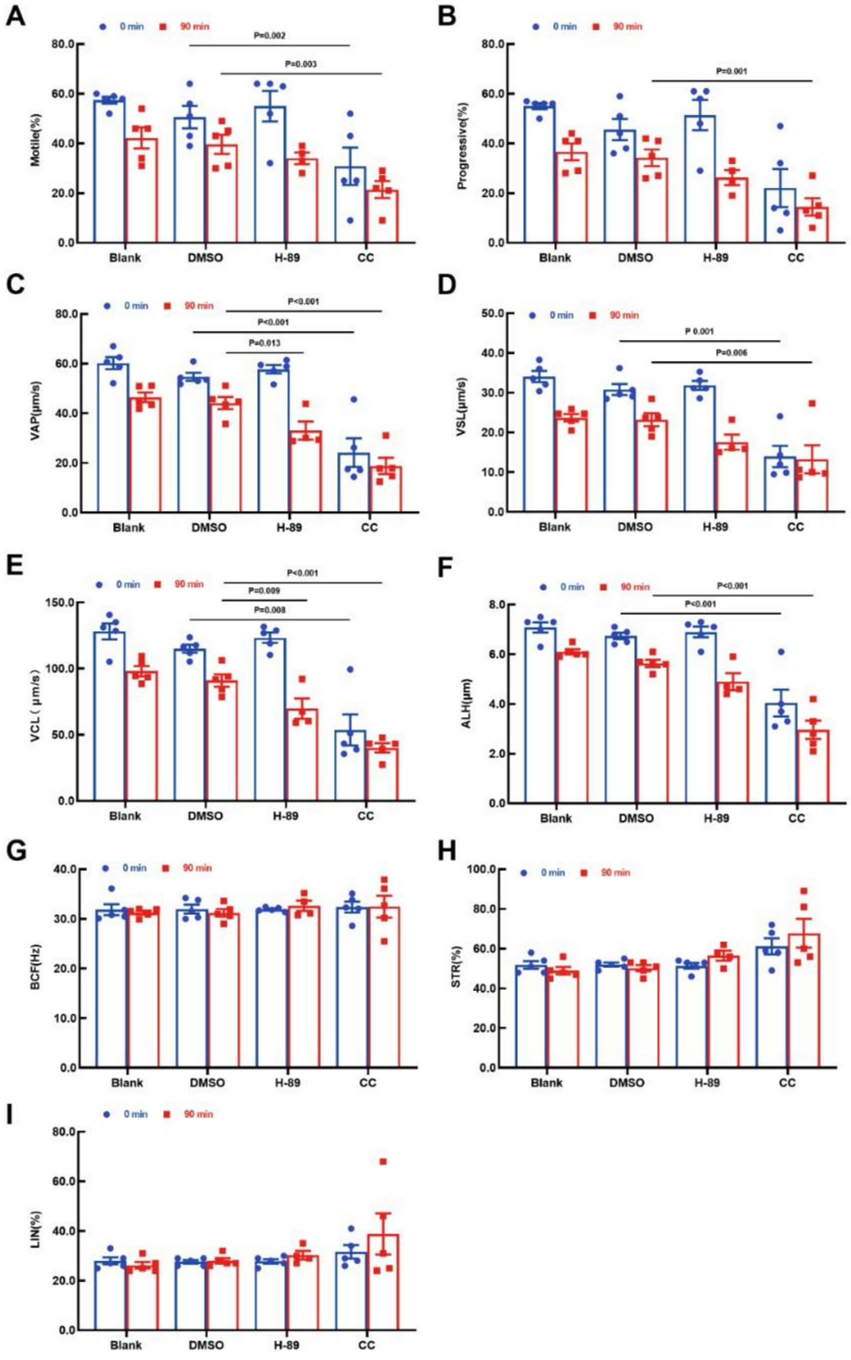

Supplement: Supplementary file 2 — Supplementary Material 2 [file 40659_2024_580_MOESM2_ESM.pdf]
