# Supplementary material for: Protein palmitoylation is involved in regulating mouse sperm motility via the signals of calcium, protein tyrosine phosphorylation and reactive oxygen species
Source: Biol Res. 2025 Jan 15;58:3. doi: 10.1186/s40659-024-00580-4 (PMC11734517; doi:10.1186/s40659-024-00580-4)

Additional File 3.

Calcium regulates sperm motility and motion parameters.

To explore whether intracellular and extracellular calcium regulates sperm motility, we carried out the CASA analysis of sperm after sperm were treated with 20 µM BAPTA-AM, intracellular calcium chelator, and 3 mM EGTA extracellular calcium chelator, respectively. The results showed EGTA decreased the percentage of progressive sperm (Figure B), VAP (Figure C), VSL (Figure D), VCL (Figure E), and ALH (Figure F), and BAPTA-AM decreased the percentage of motile sperm (Figure A), the percentage of progressive sperm (Figure B), VAP (Figure C), VSL (Figure D), VCL (Figure E), ALH (Figure F), and STR (Figure H). The results suggest that intracellular and extracellular calcium regulate sperm motility, respectively. VAP, Average path velocity; VSL, straight-line velocity; VCL, curvilinear velocity; STR, straightness (STR = VSL/VAP × 100); LIN, linearity (LIN = VSL/VCL × 100); ALH, the amplitude of lateral head displacement; BCF: beat-cross frequency. The data are presented as mean ± SEM (n=6). DMSO group, as vehicle control; $P < 0.05$, set as a statistical significance.

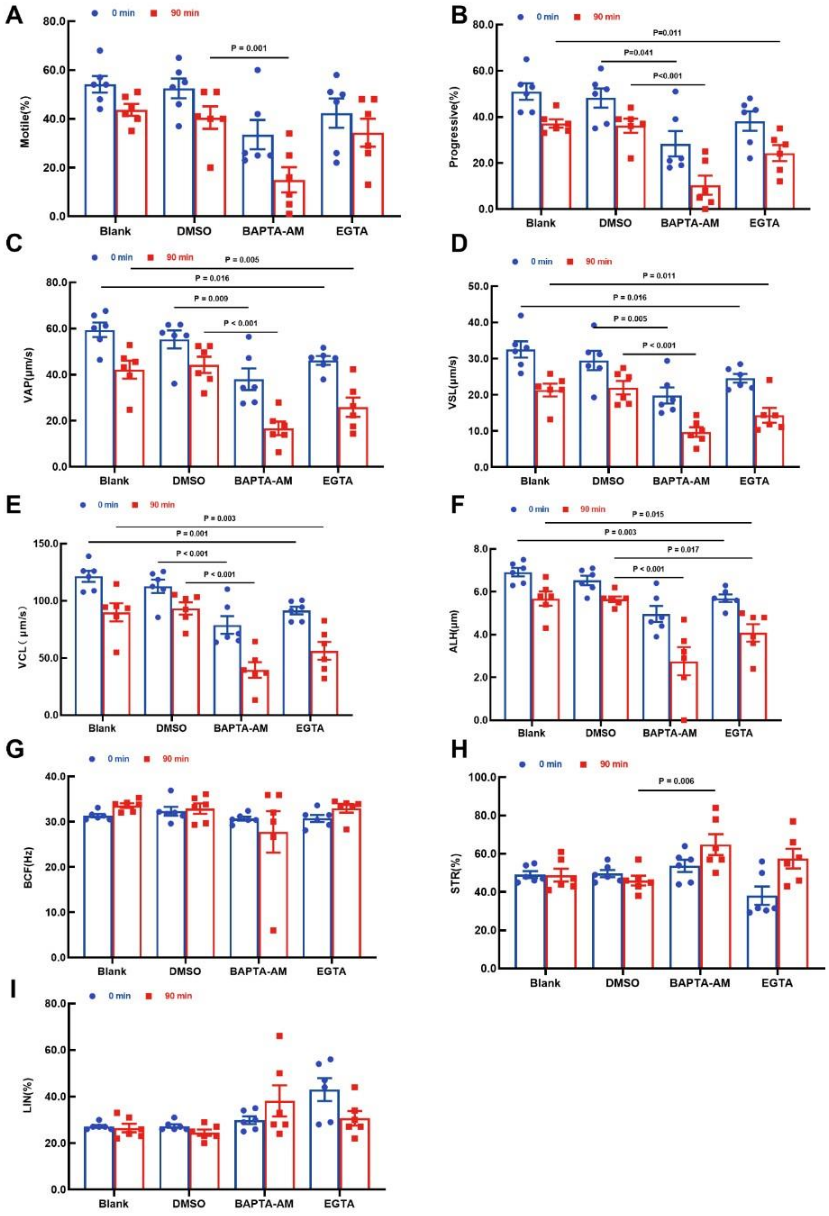

Supplement: Supplementary file 3 — Supplementary Material 3 [file 40659_2024_580_MOESM3_ESM.pdf]
